# Biological Features and Prognostic Impact of Bone Marrow Infiltration in Patients with Diffuse Large B-cell Lymphoma

**DOI:** 10.3390/cancers12020474

**Published:** 2020-02-18

**Authors:** Sara Alonso-Álvarez, Miguel Alcoceba, María García-Álvarez, Oscar Blanco, Marta Rodríguez, Mónica Baile, Juan Carlos Caballero, Julio Dávila, María Belén Vidriales, Carmen Esteban, Piedad Arias, Luis G. Díaz, Pilar Tamayo, María Dolores Caballero, Norma C. Gutiérrez, Marcos González, Alejandro Martín

**Affiliations:** 1 Department of Hematology, University Hospital of Salamanca (HUS-IBSAL), CIBERONC, and Cancer Research Institute of Salamanca-IBMCC (CSIC-USAL University), 37007 Salamanca, Spain; 2 Department of Pathology, University Hospital of Salamanca (HUS/IBSAL), 37007 Salamanca, Spain; 3 Department of General and Gastrointestinal Surgery, University Hospital of Salamanca (HUS/IBSAL), 37007 Salamanca, Spain; 4 Department of Radiology, University Hospital of Salamanca (HUS/IBSAL), 37007 Salamanca, Spain; 5 Department of Nuclear Medicine, University Hospital of Salamanca (HUS/IBSAL), 37007 Salamanca, Spain

**Keywords:** Bone marrow involvement, Diffuse large B-cell lymphoma, Discordant bone marrow, Concordant bone marrow, CNS relapse

## Abstract

The biology and clinical impact of bone marrow (BM) infiltration in patients with diffuse large B-cell lymphoma (DLBCL) remains unclear in the rituximab era. We retrospectively analyzed 232 patients diagnosed with DLBCL at our center between 1999 and 2014. Concordant-presence of large cells similar to those of the lymph node biopsy- and discordant-infiltration by small cells forming lymphoid aggregates, lacking cytological atypia-BM infiltration was defined by histological criteria and further characterized by flow cytometry (FCM). Cell of origin (COO) was determined using Hans’ algorithm. For the clonal relationship between tumor and discordant BM, the VDJH rearrangement was analyzed. Survival analyses were restricted to 189 patients treated with rituximab and chemotherapy. Thirty-six (16%) had concordant, and 37 (16%) discordant BM infiltration. FCM described different indolent lymphomas among discordant cases, clonally related with DLBCL in 10/13 available samples. Median follow-up was 58 months. 5-year-progression-free survival (PFS) for non-infiltrated, discordant and concordant groups was 68%, 65% and 30%, respectively (*p* < 0.001). Combining COO and BM infiltration, patients with discordant BM and non-germinal center B-cell COO also had decreased 5-year-PFS (41.9%). In multivariate analysis, concordant BM had an independent effect on PFS (HR 2.5, *p* = 0.01). Five-year cumulative incidence of central nervous system (CNS) relapse was 21%, 4% and 1% in concordant, discordant and non-infiltrated groups, respectively (*p* < 0.001). In conclusion, concordant BM infiltration represents a subset with poor prognosis, whereas the prognostic impact of discordant BM infiltration could be limited to non-CGB cases.

## 1. Introduction

Bone marrow (BM) is involved in 11–25% of patients diagnosed with diffuse large B-cell lymphoma (DLBCL) [1,2,3,4]. It is well known that this involvement is not always concordant with the aggressive lymphoma histology observed in the diagnostic tumor sample [5]. Several studies indicate that low-grade histology in BM (discordant BM) is present in 30–50% of DLBCL patients with BM infiltration [1,2,3,4]. Previous work has shown the unfavorable prognosis determined by a concordant marrow infiltration, with lower progression free survival (PFS) and overall survival (OS) [1,2,3,4,6]. The prognostic role of discordant BM infiltration is unclear, but it seems to be less unfavorable than concordant involvement, as no study has proven that discordant BM is an independent prognostic factor for OS. In addition, the role of BM involvement as a risk factor for central nervous system (CNS) relapse is controversial in the rituximab era [7,8].

Some studies have incorporated cytomorphological, immunophenotypic and molecular analysis, including copy number variation analysis, to improve the sensitivity of the tumor infiltrate detection, and to provide a detailed biological description [1,9,10]. However, little information is available regarding the biological mechanisms involved in the development of both types of infiltrate. Furthermore, it is not known whether there is any relation between the type of infiltration and the cell of origin of DLBCL. In addition, the possible clonal relationship between the tumor and BM clones in discordant cases has barely been studied [9]. With the little genetic data available, we cannot confirm whether the relation between low-grade and high-grade histologies is similar to what has been described in other transformation models.

On this basis, we retrospectively analyzed our series of patients diagnosed with DLBCL to examine the prognostic impact and biological features of discordant and concordant BM infiltration. We included flow cytometry (FCM) analysis of the diagnostic BM aspirates, to improve the sensitivity of tumor detection and to characterize phenotypically low-grade infiltrates. We also questioned the relationship of the cell of origin (COO) of the aggressive tumor sample and the type of BM infiltration. In addition, we analyzed the possible clonal relationship between the tumor and BM clones in discordant cases.

## 2. Patients and Methods

### 2.1. Study Design and Patients

This is a retrospective observational study performed in the Hospital Universitario de Salamanca. We identified patients by reviewing Hematology and Pathology Department databases, and selecting those cases with a histological diagnosis of DLBCL. We included all consecutively diagnosed patients between 1999 and 2014 with staging BM biopsy. Those with DLBCL transformed from previous indolent lymphoma and primary central nervous system lymphomas were excluded. The study was approved by our institutional ethics committee (code: “PI 2016 11 001”).

### 2.2. Histopathological Review and Immunohistochemistry

An expert hemopathologist reviewed all of the BM core biopsies for the present study. Concordant and discordant BM infiltration was defined by histological criteria. Concordant involvement was defined as the presence of large cells, similar to those present in the initial lymph node diagnostic biopsy. Discordant involvement was defined as infiltration by small cells forming lymphoid aggregates, lacking cytological atypia. In some cases, the pathologist reported an unspecific lymphoid proliferation not suggestive of malignancy; in those cases, flow cytometry (FCM) was used to characterize the infiltrates further, and whether a clonal B-cell population of any size was found by FCM, these were included in the discordant group.

The cell of origin (COO) was determined in the original tumor sample using immunohistochemistry (IHC), according to the algorithm of Hans et al [11]. DLBCL cases were classified as germinal center B-cell-like (GCB) or non-GCB.

### 2.3. Flow Cytometry Analysis

BM aspirates obtained in the same procedure as the BM biopsies were analyzed by FCM, and when available, were sent to the Molecular Biology and Cytogenetic Laboratories as part of the routine extension study for DLBCL.

BM aspirates were processed according to conventional procedures [12,13]. Samples were stained using a four-color (from 1999 to 2010) or eight-color (from 2011 to 2014) direct immunofluorescence technique. For four-color protocols, the following monoclonal antibody (MoAb) combinations (FITC/PE/PerCP-Cy5.5/APC) were used for screening: FMC7/CD5/CD19/CD45, and sK/sL/CD19/CD20. For eight-color protocols, the MoAb combinations (FITC/PE/PerCP-Cy5.5/PE-Cy7/APC/APC-H7/BV450/OC515) used were: sK/sL/CD5/CD19/ CD10/ CD38/CD20/CD45.

After staining, samples were acquired on a FACSCalibur or FACSCanto-II flow cytometer (Becton/Dickinson Biosciences (BD), San José, CA, USA), using CellQUEST or DIVA software (BD), respectively. For the analysis, the Paint-a-Gate (Becton Dickinson Biosciences) or INFINICYT™ software programs (Cytognos SL, Salamanca, Spain) were used.

### 2.4. Clonality Analysis and IGHV Gene Sequencing

Tumor DNA was extracted from samples collected at the time of diagnosis. In fresh samples, high-molecular-weight DNA was isolated using DNAzol reagent (MRC, Cincinnati, OH, USA). Clonality assessment and immunoglobulin heavy-chain variable region (*IGHV*) genes rearrangement were analyzed in both lymph node and bone marrow samples from the group of patients with discordant BM involvement (histologically and/or phenotypically documented). Tumor samples were tested for the amplification of *IGH* rearrangements according to the BIOMED-2 Concerted Action protocols [14]. Complete V–D–J rearrangement amplification was performed by multiplex PCR with a set of family-specific primers of the framework region 1 (FR1) and one IGHJ consensus primer. For the samples with no detectable amplification from FR1, PCR was performed with the FR2 region. The presence of the monoclonal rearrangement was confirmed by GeneScan with using ABI 3130xL or ABI 3500xL DNA Sequencers (Applied Biosystems, Foster City, California). PCR products were then sequenced directly, using Big-Dye terminators (Applied Biosystems). Germline *IGHV* genes from complete V-D-J rearrangements were identified using the IMGT/V-QUEST database (http://www.imgt.org, last accessed November 18 2016). We recorded *IGHV* gene usage, and the percentage of *IGHV* identity to the closest germline gene.

### 2.5. Fluorescence in Situ Hybridization Analysis

Interphase fluorescence in-situ hybridization (FISH) studies were carried out in the group of DLBCL with involved bone marrow. Carnoy fixed cells preserved at diagnosis of lymph node samples were used in most patients, although in those concordant cases without lymph node available, the infiltrated BMs were used. *MYC* and *BCL6* rearrangements, t(14;18)(q32;q21) *(BCL2)* and 17p13.1 region *(TP53)* were explored by using the FISH probes, “LSI *MYC* and LSI *BCL6* Dual Color Break Apart Rearrangement”, “*IGH/BCL2* Dual Color Dual Fusion Translocation” and “LSI *TP53*” acquired from Abbott Molecular (Des Plaines, IL) following the manufacturer’s recommendations.

### 2.6. Endpoints and Statistical Methods

Primary endpoints were i) PFS, calculated from the date of diagnosis until the date of relapse, progression, death from any cause, or last follow-up; ii) cumulative incidence of CNS relapse, documented by FCM or image techniques (computed tomography and/or magnetic resonance imaging) when clinically suggestive; and iii) OS, calculated from the date of diagnosis until the date of death from any cause or last follow-up.

Survival analyses was only performed among the group of patients (n = 189) who had been homogeneously treated with rituximab plus chemotherapy with a curative intent. Clinical data were last updated on November 18 2016. The main clinical–biological variables at diagnosis were abstracted, including the date of birth, date of DLBCL diagnosis, revised International Prognostic Index (R-IPI) [15] variables and score, the involvement of other extranodal sites, CNS-IPI score [16], first-line therapy, progression, death and date of last follow-up. All patients were staged at diagnosis with computed tomography and bone marrow biopsy.

We considered death as a competing risk to calculate the cumulative incidence of CNS relapse. Cumulative incidence curves were plotted, and Gray’s test was used to calculate the statistical differences, using R software (available at www.r-project.org). Survival curves were plotted using the Kaplan–Meier method, and the differences were analyzed using the log-rank or Breslow test, as appropriate. Multivariate analysis was carried out using the time-dependent Cox-proportional hazard model, including all variables with some indication of a significant association in the univariate test (*p* < 0.1) or those considered relevant in previous reports. Group means were compared using Student’s t-test. Qualitative variables were analyzed by the Pearson χ^2^ test or Fisher’s exact test, as appropriate. Two-tailed values of *p* < 0.05 were considered significant. All analyses were carried out with SPSS, v.20 (IBM, Armonk, NY, USA).

## 3. Results

### 3.1. The Combination of Histology and Flow Cytometry Improved the Bone Marrow Involvement Classification

A total of 232 patients sequentially diagnosed with DLBCL in our center between January 1 1999 and December 31 2014 were included. According to their histology, 57 patients (25%) had BM infiltration, of which 36 (16%) were concordant, and 21 (9%) discordant. FCM analysis revealed lymphoma infiltration in 16 (7%) additional patients with unspecific lymphoid infiltrates, and were included in the discordant group. Clinical characteristics of patients are summarized in Table 1. As shown in Table 1, poor-risk (R-IPI) was more frequent among patients in the concordant group (85%) than in the discordant (59%) or non-infiltrated (41%) groups (*p* < 0.001).

FCM results in BM were available in 35 out of 36 patients with concordant BM infiltration, and all 21 patients with discordant BM infiltration (Table 2). In concordant cases, FCM results were suggestive of DLBCL in most patients. In contrast, FCM showed a wide variety of indolent B-cell lymphomas in the discordant cases (Table 2). FCM did not show tumor infiltration in 14 cases with discordant (n = 4) or concordant (n = 10) histological BM infiltration, probably due to the type of infiltration, which, in all cases, was either focal or interstitial.

We found no statistically significant associations between the cell of origin of the DLBCL and the type of BM infiltration. Thus, 64% of concordant cases were non-GCB-DLBCL while 62% of discordant cases, and 63% in the non-infiltrated BM group (*p* = 1).

### 3.2. Clonality Studies Revealed a Unique Clone in most of the Discordant Cases

We had 18 paired lymph node and BM samples from the group of discordant patients (histologically and/or phenotypically documented). A polyclonal trace was observed in at least one sample in five cases, and therefore the clonality relationship was undetermined. A clonal peak could be identified in 13 cases, which revealed a common monoclonal peak in 10 patients, and different clones in three patients, all of them with a CLL phenotype. We sequenced *IGHV* genes in those patients with a common clonal origin. The genes most frequently used were *IGHV4-34* (n = 2) and *IGHV3-48* (n = 2), and there were six individual cases for *IGHV1-3*, *IGHV1-8*, *IGHV3-7*, *IGHV3-33*, *IGHV3-53* and *IGHV3-72*, respectively.

### 3.3. FISH Analysis Did Not Find Different Genetic Abnormalities between Concordant and Discordant Groups

Twenty-one patients, 14 with concordant BM infiltration and 7 with discordant infiltration, could be analyzed by FISH (Table 3). Most of the patients with concordant and discordant BM showed genetic aberrations in one or more of the analyzed genes (64% in concordant BM, and 86% in discordant BM group, *p* = NS). Interestingly, discordant BM cases showed gains in *MYC* more frequently than concordant BM (57% vs. 14%, *p* = 0.06). No other genetic alteration differs between BM involvement groups.

*BCL2* alterations were differentially distributed between COO groups. Thus, *BCL2* translocations were more frequently found in GCB-DLBCL (70% in GCB-DLBCL vs. 9% in non-GCB, *p* = 0.007), while *BCL2* gains were only found in non-GCB-DLBCL (54% vs. 0%, *p* < 0.001), independently of the type of BM infiltration. Double-hit translocations affecting *BCL2* and *MYC* (n = 2) and triple-hit translocations affecting *BCL2*, *MYC* and *BCL6* (n = 1) were only observed in the GCB group. Interestingly, one patient showed a double hit translocation (*BCL2* and *MYC*), and was the only one with discordant BM and GCB COO that progressed after rituximab plus curative chemotherapy.

### 3.4. Concordant BM Cases Displayed Shorter PFS and Higher Incidence of CNS Relapse

Survival was only analyzed in the 189 patients treated with rituximab plus chemotherapy with curative intent (R-CHOP [rituximab, cyclophosphamide, doxorubicin, vincristine, prednisone] n = 152 (80.4%), R-MegaCHOP n = 22 (11.6%), R-COMP [pegylated doxorubicin instead doxorubicin] n = 5 (2.6%), VR-CAP [bortezomib instead vincristine] n = 4 (2.1%) and others n = 6 (3.2%)). Nine of 31 patients (29%) in the discordant BM group and 13 of 35 patients (37%) in the concordant group received prophylaxis for CNS relapse, consisting of 4–6 doses of triple intrathecal therapy (methotrexate 12 mg, cytarabine 30 mg and hydrocortisone 20 mg). Median follow-up of surviving patients was 58 months (1–152 months).

**Figure 1:**
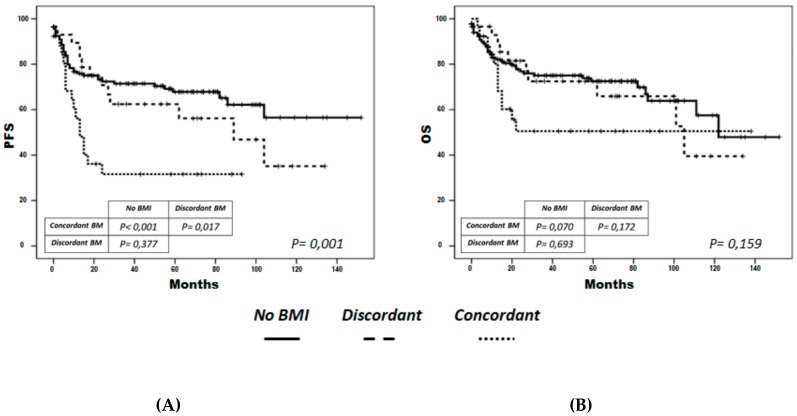
Progression-free survival (PFS) (**A**) and overall survival (OS) (**B**) by bone-marrow infiltration type. PFS and OS were significantly lower (*p* < 0.001 and *p* = 0.08, respectively) in patients with concordant infiltration (dotted line), than in those without BM infiltration (continuous line) and with discordant infiltration (discontinuous line). No significant differences were observed between the latter two groups (p>0.1).

PFS at 5 years was 68% for the non-infiltrated group, 62% for the discordant group (p = 0.4) and 32% for the concordant group (*p* < 0.001) (Figure 1A). In the multivariate analysis including COO and R-IPI, concordant BM infiltration had an independent impact on PFS, over and above that of the R-IPI as a global score (HR = 2.2; 95% CI = 1.1-4.3; *p* = 0.02) (Table 4), when categorized according to risk groups, or when testing the individual variable content in the R-IPI separately (results not shown). 

Patients with concordant BM had a lower 5-year OS (51%) than those in the discordant (72.5%) and non-infiltrated (72.5%) groups (*p* = 0.06) (Figure 1B), although the only variable with independent impact on OS was the R-IPI (Table 4). In the concordant group, 6 out of 18 patients with progressive disease received autologous stem-cell transplantation as salvage therapy, and two of them needed to be rescued afterwards with allogeneic stem-cell transplantation (allo-SCT). Four of them, including both who received allo-SCT, were disease-free at the time of writing.

We have reanalyzed PFS and OS, only considering those patients with stage IV in order to assess the prognostic impact of BM infiltration in comparison with other extranodal sites. In this setting, PFS at 5 years was 63% for the non-infiltrated group, 62% for the discordant group and 32% for the concordant group (*p* < 0.05, Figure S1A). There were no statistically significant differences in terms of 5-year OS in concordant BM (51%) as compared to discordant (72.5%) and non-infiltrated (63%) groups (*p* = 0.5) (Figure S1B).

**Figure 2:**
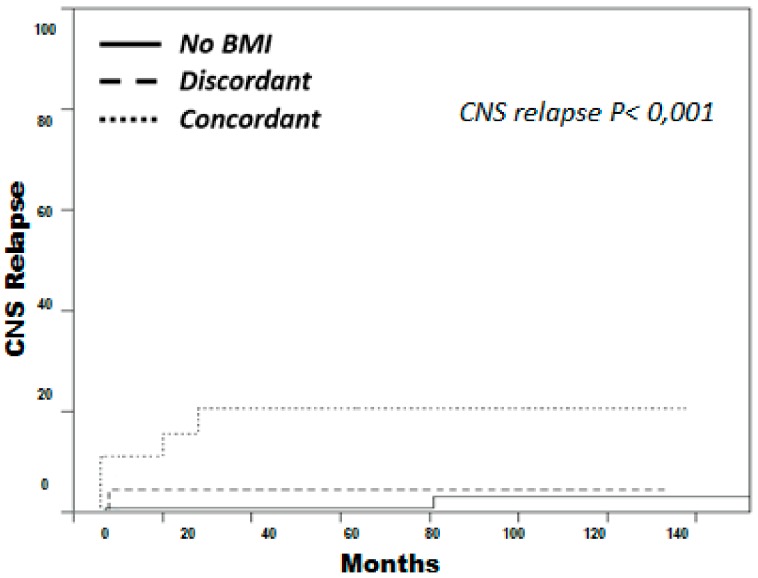
Cumulative incidence of central nervous system (CNS) relapse by bone-marrow infiltration type. Considering death as a potential competing risk, cumulative incidence of CNS relapse was significantly higher in the concordant group (dotted line) as compared to the discordant group (discontinuous line) and the non-infiltrated group (continuous line).

Five-year cumulative incidence of CNS relapse was significantly higher in the concordant group (21%) as compared to the discordant group (4%) and the non-infiltrated group (1%, *p* < 0.001) (Figure 2). In the multivariate analysis, including the type of BM infiltration and the variables included in CNS-IPI, concordant infiltration had an independent association with CNS relapse (HR: 10.1; 95% CI: 2.2–46.3; *p* = 0.003) (Table 5).

### 3.5. Non-GCB discordant BM Cases Could Display Shorter PFS

**Figure 3:**
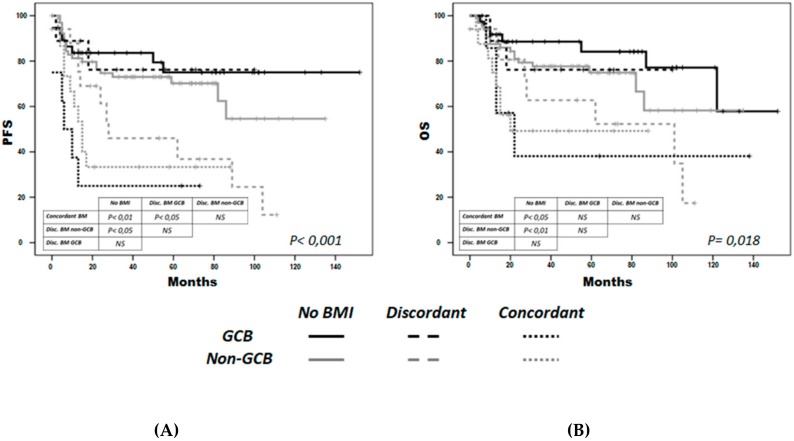
Progression-free survival (PFS) (**A**) and overall survival (OS) (**B**) by bone-marrow infiltration type and cell of origin. Concordant BMI was represented as a dotted line, discordant BMI as a discontinuous line, and the non-infiltrated BMI as continuous line. Black lines represent germinal center B-cell like (GCB), while gray non-GCB DLBCL. (**A**) PFS was significantly decreased in both concordant BMI groups as compared to both non-infiltrated groups, and discordant GCB group (*p* < 0.05, all comparisons). PFS was also lower in the discordant non-GCB group than in the non-infiltrated groups (*p* < 0.05). (**B**) OS was significantly lower in both concordant groups, and in the discordant non-CGB group, compared with the non-infiltrated groups (*p* < 0.05).

By combining the type of BM infiltration and COO, we observed that both concordant non-GCB (n = 16) and GCB (n = 8) patients had a lower 5-year PFS (33% and 25%, respectively) than the non-infiltrated GCB (n = 32) and non-GCB (n = 64) groups (78.5% and 70%, respectively), and the discordant GCB (n = 9) group (76%) (*p* < 0.001 for all comparisons). Notably, 5-year PFS in the discordant non-GCB group (n = 17) was 46%, a significantly worse figure than in the non-infiltrated group (Figure 3A). In the multivariate analysis, concordant GCB and non-GCB groups had a higher risk of progression independently of R-IPI; the higher risk in the discordant non-CBG group was close to statistical significance (Table 4).

Similarly, concordant GCB and non-GCB groups and the discordant non-GCB group had significantly lower OS (38%, 49% and 63% at 5 years, respectively) than the discordant GCB, non-infiltrated GCB and non-GCB groups (76%, 81% and 75%, respectively, (*p* < 0.05) (Figure 3B), although the association was not independent of the R-IPI in the multivariate analysis (Table 4).

## 4. Discussion

The use of highly effective rituximab-containing primary therapy in DLBCL makes it more difficult to salvage patients who are refractory or who relapse [17]. Therefore, early recognition of poor prognostic patients is very important for exploring alternative first-line treatment strategies. In our series of 189 patients treated with R-CHOP-like, we observed that high-grade BM involvement had a significantly unfavorable effect on PFS, independent of R-IPI, even considering only stage IV patients. This finding is in accordance with the results reported by Sehn et al. [2] from larger series and, in agreement with these observations, a recent study published by Yao et al. [18] has suggested that patients with DLBCL and concordant BM involvement should be considered a distinct entity due to a more aggressive course and poor prognosis in terms of both PFS and OS. However, in our series, concordant involvement had no independent prognostic influence on OS in the multivariate analysis, probably due to the small number of patients with concordant infiltration and to the relatively high efficacy of the salvage therapies employed, including autologous and allogeneic stem cell transplantation.

Another relevant finding was the high incidence of CNS relapse in the concordant BM patients in our series. BM involvement has been cited as a risk factor for CNS involvement [8], although a study performed in the rituximab era found that BM involvement was associated with a higher rate of CNS relapse only in the presence of increased LDH [7]. In our study, concordant, but not discordant, BM involvement was a risk factor for CNS relapse, independent of CNS-IPI. This finding has been previously described by Sehn et al. in another retrospective analysis [2]. In both studies, the number of events was small and the multivariate analysis was limited to the IPI or CNS-IPI score, so larger studies are needed to confirm these results. In the recent and previously mentioned analysis from Yao et al. [18], investigators do not describe a higher incidence of CNS relapse in patients with concordant BM infiltration. However, CNS prophylaxis, including systemic high dose methotrexate (MTX) and cytarabine or intrathecal MTX, were administered to 61.8% of patients. In our series, CNS prophylaxis was administered only to 26% of patients with concordant BM infiltration, and it was only based on intrathecal triple therapy (MTX, cytarabine and hydrocortisone).

Therefore, although based upon retrospective data, as it is the case of the majority of studies that analyze the risk factors for CNS relapse [19], our results suggest that patients with concordant BM involvement are candidates for CNS evaluation and systemic CNS prophylaxis.

We explored the biological characteristics of patients with discordant BM infiltration in order to understand better their unclear prognosis. As Brudno et al. noted in their review [5], there is a paucity of published information about the biology, diagnosis, treatment and outcome of discordant lymphomas. First of all, we observed that although COO is not related to the type of infiltration, it gains relevance as a potential prognostic factor when combined with the type of BM involvement. Patients who have both discordant BM and non-GCB-DLBCL have a significantly lower PFS, the association being close to statistical significance in the multivariate analysis. To our knowledge, this finding has not been previously reported. In contrast, discordant BM in patients with GCB-DLBCL had similar outcomes to those without BM infiltration in our series of patients, which questions whether these cases should be considered stage IV. This might explain the lack of consensus about discordant DLBCL outcomes in previous studies. However, due to the small sample size of subgroups, these findings should be interpreted with caution, and should be confirmed in larger series and, preferably, with COO determination based on gene expression profile methods.

So far, FCM has not demonstrated clinical value in determining the prognosis of patients with DLBCL, but it is helpful in the phenotypic characterization Thus, in accordance with previous studies [9], we observed that FCM helps both to define the type of low-grade lymphoma observed in the BM, and to enhance the sensitivity of histology to detect BM involvement. Nowadays, when bicrestal biopsies are not commonly performed, FCM might be useful in addition to bone marrow trephine in detecting BM involvement [20]. In our series, FCM was beneficial mainly for characterizing unspecific patterns of infiltration for which there is no conclusive evidence of lymphoma, but also aided the phenotypic description of discordant cases. With the new techniques of high-sensitivity FCM and the progressive standardization of protocols [21], the detection of BM infiltration in patients with lymphoma is likely to improve, so prospective studies incorporating these new techniques are needed to assess the real clinical value of FCM in DLBCL patients.

Several studies have suggested that FDG PET can predict BM infiltration with high sensitivity in aggressive non-Hodgkin lymphomas [22,23]. However, PET is less sensitive at detecting low-grade infiltrates [24], which account for a notable percentage of the BM infiltrations in patients with DLBCL. Although identification of high-grade BM infiltration seems to be critical for determining the prognosis, detection of low-grade infiltrates could also have prognostic value when combined with the COO, as mentioned before. So, the BM biopsy and aspirate might still be needed, at least in prospective studies, to better define the genuine prognostic impact of discordant involvement.

Clonality studies analyzing the relation between low-grade and high-grade lymphoma in patients with discordant BM demonstrated a very high rate of clonal identity, as previous studies have suggested [9]. These could be interpreted as potential cases of transformation from different subtypes of indolent lymphoma. Interestingly, three out of the five discordant BM cases with a CLL phenotype were not clonally related. Genomic analysis may be necessary to determine whether the mechanisms involved in transformation in this group of patients match those observed in a series of documented high-grade transformations from the different indolent lymphomas [25,26,27,28].

In our series, we found numerous genetic aberrations involving *MYC*, *TP53*, *BCL2* and *BCL6* in the patients with BM involvement. Particularly, we have documented a high incidence (70%) of (14;18) translocations, affecting the anti-apoptotic gene *BCL2*, in the group of patients with BM infiltration and GCB-DLBCL subtype, while the frequency reported in GCB-DLBCL, irrespective of BM infiltration, is 18%–35% [29,30]. This might explain in part the bad prognosis of this group of patients due to the clinical role of this gene in patients with DLBCL in other studies [29,30,31,32]. *MYC* gains were more frequently observed in the group with discordant BM, and it was equally distributed between both COO groups. The clinical significance of *MYC* gains remains controversial [33], although recent reports suggest a worse clinical outcome in GCB-DLBCL [31].

*TP53* deletion, and gains and translocations of *BCL6*, were homogenously distributed in the group of patients with infiltrated BM, without any bias for any of the groups, probably due to the reduced number of patients included.

## 5. Conclusions

In conclusion, our results show that concordant BM infiltration was associated with lower PFS and a high incidence of CNS relapse independent of COO and IPI, suggesting that different treatments from R-CHOP are needed for these patients. Discordant BM infiltration is clonally related to the aggressive histology in the majority of cases, and transformation mechanisms should be analyzed in future studies. In a different way to the concordant infiltration, the prognostic impact of discordant BM infiltration could be limited to the non-CGB cases. Although based on a single-center retrospective analysis with a limited number of cases, our results are intriguing, and should be confirmed in larger and prospective series. The incorporation of new, highly sensitive techniques, such as FCM and PET, as well as extensive genomic studies, will contribute in the future to the better diagnosis, characterization and prognostic evaluation of BM infiltration in patients with DLBCL.

## Figures and Tables

**Table 1:** Patient characteristics according to the bone marrow infiltration.

Variable	Non-infiltrated BM (n = 159)	Discordant BM (n = 37)	Concordant BM (n = 36)	*p*
**Gender (Male)**	82 (52%)	12 (32%)	20 (56%)	0.07
**Age > 60 years**	106 (67%)	27 (73%)	20 (56%)	0.3
**Ann Arbor stage > 2**	91 (58%)	37 (100%)	36 (100%)	<0.001
LDH (high) *	50 (39%)	13 (41%)	20 (77%)	0.0018
Extranodal sites > 1 *	27 (20%)	16 (48%)	19 (68%)	<0.001
ECOG ≥ 2 *	41 (32%)	8 (25%)	14 (54%)	0.05
R-IPI > 2 *	54 (41%)	20 (59%)	22 (85%)	<0.001
COO non-GCB *	64 (63%)	19 (62%)	19 (64%)	0.99

* In the descriptive analysis, either ECOG, R-IPI, LDH or extranodal sites data was not available in 32 non-infiltrated BM, 5 discordant BM, and 10 concordant BM; COO was not available in 58, 7 and 6 cases, respectively. Regarding patients included in the survival analysis (n = 189), either ECOG, R-IPI, LDH or extranodal sites data were not available in 9/134 patients with non-infiltrated BM, 2/26 with discordant BM and 1/29 with concordant BM, while COO was not available in 33, 3 and 2 cases, respectively. Abbreviations: BM: bone marrow; COO: Cell-of-origin; R-IPI: revised International Prognostic Index.

**Table 2:** Phenotypic characteristics of bone marrow (BM) lymphoid infiltrates according to histology.

Histology	FCM							
	DLBCL	DLBCL+FL	FL	CLL	MZL-like	LPL-like	NS	Non-Infiltrated
High-grade (Concordant BM, n = 35)	21	−	3	−	−	−	1	10
Low-grade (Discordant BM, n = 21)	1	1	3	4	2	−	6	4
Unspecified lymphoid infiltrate (n = 31) *	1	−	4	4	2	1	4	15

* The 16 cases with a clonal B-cell population of any size found by FCM were included in the discordant group. Abbreviations: CLL: chronic lymphocytic leukemia; DLBCL: diffuse large B-cell lymphoma; FL: follicular lymphoma; MZL: marginal zone lymphoma. LPL: lymphoplasmacytic lymphoma. NS: B-cell NHL with non-specific phenotype.

**Table 3:** Fluorescence in-situ hybridization (FISH) analysis of patients with involved bone marrow.

UPN	Sample	% FCM	(14;18) (*IGH/BCL2*)	8q24 (*MYC*)	3q26 (*BCL6*)	17p13.1 (*TP53*)	COO	BM
3470	LN	60	**Clonal**	**Clonal**	**Clonal**	Normal	GCB	C
7062	LN	9,5	**Clonal**	Normal	Normal	Normal	GCB	C
4512	BM	21	Normal	Normal	Normal	Normal	GCB	C
3999	LN	12	Normal	Normal	Normal	Normal	GCB	C
9102	BM	15	**Clonal**	Normal	**Gain**	Normal	GCB	C
9225	LN	66,7	**Clonal**	**Clonal**	Normal	Normal	GCB	C
2704	BM	15	**Gain**	Normal	Normal	Normal	Non-GCB	C
3985 ^*^	LN	47	**Gain**	**Gain**	**Gain**	**Gain**	Non-GCB	C
6908	LN	15	Normal	Normal	Normal	Normal	Non-GCB	C
6156 ^†^	LN	*NA*	**Gain**	**Gain**	**Gain**	**Gain**	Non-GCB	C
7288	LN	69	**Gain**	Normal	*NS*	Normal	Non-GCB	C
5296	LN	15	Normal	Normal	Normal	Normal	Non-GCB	C
8275	BM	36,6	Normal	Normal	Normal	Normal	Non-GCB	C
9043	LN–FFPE	*NA*	**Gain**	**Clonal**	Normal	*NS*	Non-GCB	C
4859	LN	*NA*	**Clonal**	**Gain**	Normal	Normal	GCB	D
4989	LN	75	**Clonal**	Normal	**Clonal**	Normal	GCB	D
7029	LN	78	Normal	**Gain**	**Gain**	Normal	GCB	D
8724 ^*^	LN	93	**Clonal**	**Clonal**	**Gain**	**Gain**	GCB	D
3671	LN	60	Normal	Normal	Normal	Normal	Non-GCB	D
8818	LN	48	**Clonal**	**Gain**	Normal	Normal	Non-GCB	D
9386	LN	64	**Gain**	**Gain**	**Gain**	**Deletion**	Non-GCB	D

* Tetraploid karyotype; † Hyperdiploid karyotype. Abbreviations: BM: bone marrow; C: concordant infiltration; COO: cell of origin; D: discordant infiltration; FCM: Flow cytometry; FFPE: formalin-fixed paraffin-embedded; GCB: germinal center B-cell like; LN: lymph node; NA: not available.

**Table 4:** Multivariate analysis of progression-free survival and overall survival.

Variable	PFS			OS		
	HR	95% CI	*p*-Value	HR	95% CI	*p*-Value
Bone marrow infiltration	−	−	−	−	−	−
No infiltration (reference)	−	−	−	−	−	−
Concordant	2.2	1.1-4.3	0.02	1.6	0.7-3.4	0.2
Discordant	1.5	0.7-3	0.2	1.5	0.7-3.2	0.3
R-IPI score (0 to 5)	1.3	1.0-1.5	0.03	1.4	1.1-1.8	0.004
Bone marrow infiltration and COO						
No infiltration (reference)	−	−	−	−	−	−
Concordant GCB	2.9	1–8.7	0.04	1.2	0.3–4.4	0.2
Concordant Non-GCB	3	1.4–6.4	0.003	1.6	0.7–3.9	0.3
Discordant GCB	0.7	0.1–3	0.6	0.7	0.1–3.4	0.7
Discordant Non-GCB	1.9	0.9–4.2	0.08	1.6	0.6–3.7	0.3
R-IPI score (0 to 5)	1.2	0.9–1.5	0.1	1.5	1.2–1.9	<0.001

Abbreviations: COO: Cell of origin; GCB: germinal center B-cell like; R-IPI: revised International Prognostic Index; OS: overall survival; PFS: progression-free survival.

**Table 5:** Univariate and multivariate analysis of central nervous system relapse incidence.

Variable	Univariate		Multivariate		
	5-Year CNS Incidence	*p*-Value	*p*-Value	HR	95% CI
Bone marrow infiltration No infiltration Discordant Concordant	0.8% 7.3% 3.1%	<0.001	0.003	10.1	2.2–46.3
Renal/Adrenal involvement No Yes	3.8% 22.2%	0.007	0.017	7.8	1.5–42
Lactate dehydrogenase No Yes	3.5% 13%	0.3	−	−	−
ECOG 0–1 2–4	4.2% 10%	0.8	−	−	−
Ann Arbor stage <3 ≥3	8% 7%	0.4	−	−	−
Extranodal sites <2 ≥2	3.7% 12.5%	0.1	−	−	−
Cell of origin GCB Non-GCB	6.8% 3.5%	0.8	−	−	−

Abbreviations: CNS: central nervous system; GCB: germinal center B-cell like.

## Supplementary Materials

The following are available online at https://www.mdpi.com/2072-6694/12/2/474/s1, Figure S1: Progression-free survival (PFS) (A) and overall survival (OS) (B) by bone-marrow infiltration type considering those patients with stage IV.
