# Supplementary figures and images for: Biological Features and Prognostic Impact of Bone Marrow Infiltration in Patients with Diffuse Large B-cell Lymphoma
Source: Cancers (Basel). 2020 Feb 18;12(2):474. doi: 10.3390/cancers12020474 (PMC7072385; doi:10.3390/cancers12020474)

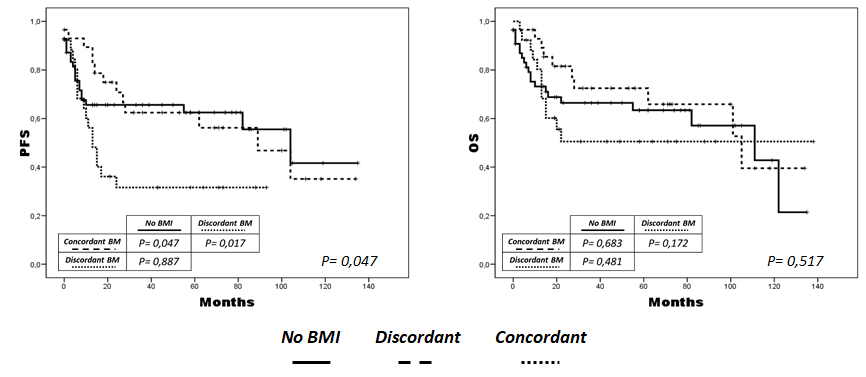

Supplement: Supplementary file 1 [file cancers-12-00474-s001.zip › Figure S1.tif]
